# Supplementary material for: Effect of the Genotypic Variation of an Aphid Host on the Endosymbiont Associations in Natural Host Populations
Source: Insects. 2021 Mar 4;12(3):217. doi: 10.3390/insects12030217 (PMC8001399; doi:10.3390/insects12030217)
Supplement: Supplementary file 1 [file insects-12-00217-s001.pdf]

Table S1 Total number of sampled live aphids and of the different localities studied.

Locality	Number of sampled aphids	GPS point
Llastuco	622	39°39’45’’S/ 73°02’22’’W
Mafil	1224	39°43’00’’S/ 73°04’11’’W
Santa-Elvira	1018	39°46’47’’S/ 73°11’39’’W
Pichoy	760	39°41’45’’S/ 73°06’27’’W

Table S2 Relative frequency of each aphid clone based in its microsatellite genotype (8 loci) sampled in the field.

Aphid clone	Relative frequency		Microsatellite genotype															
			S4Σ	S4Σ	S5L	S5L	S30	S30	S16b	S16b	Sm17	Sm17	Sm10	Sm10	Sm11	Sm11	S3.43	S3.43
G1	437	26,5%	171	171	226	228	189	191	222	222	111	111	175	181	160	170	184	186
G2	402	24,3%	175	181	243	245	177	179	225	225	115	115	185	187	160	164	203	203
G3	376	22,8%	181	185	241	245	179	179	205	205	115	115	187	187	160	164	203	212
G4	169	10,2%	181	185	241	245	177	179	190	205	115	115	185	187	160	164	203	203
G5	92	5,6%	181	185	241	245	177	177	205	205	115	115	185	185	160	160	203	212
G6	58	3,5%	181	185	241	245	177	179	205	205	115	115	185	185	160	164	203	212
G7	51	3,1%	181	185	241	243	177	179	205	205	115	115	185	187	160	160	203	212
G8	12	0,7%	175	175	241	245	177	187	213	213	115	115	0	0	164	164	203	203
G9	11	0,7%	181	185	241	245	177	179	205	205	115	115	185	187	0	0	203	212
G10	9	0,5%	175	175	241	245	177	179	205	205	115	115	172	172	160	164	182	212
G11	9	0,5%	181	185	241	243	177	177	205	205	115	115	185	185	160	164	203	212
G12	8	0,5%	175	181	243	245	177	179	225	225	115	115	185	187	164	164	203	203
G13	5	0,3%	181	185	241	245	177	179	207	225	115	115	185	185	0	0	203	212
G14	5	0,3%	181	181	243	245	176	176	190	190	115	115	0	0	160	164	203	203
G15	5	0,3%	171	171	220	222	189	191	222	222	111	111	0	0	160	170	0	0
G16	2	0,1%	175	181	241	245	177	179	205	227	115	115	0	0	0	0	203	212

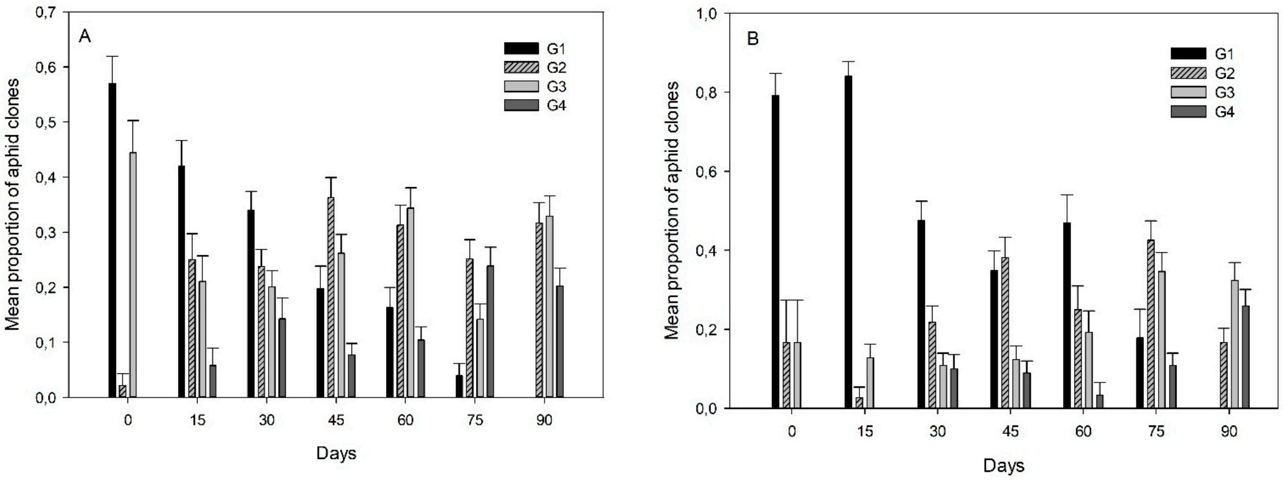

Figure S1. Predominance of common aphid clones on A) wheat and B) oat across the season.

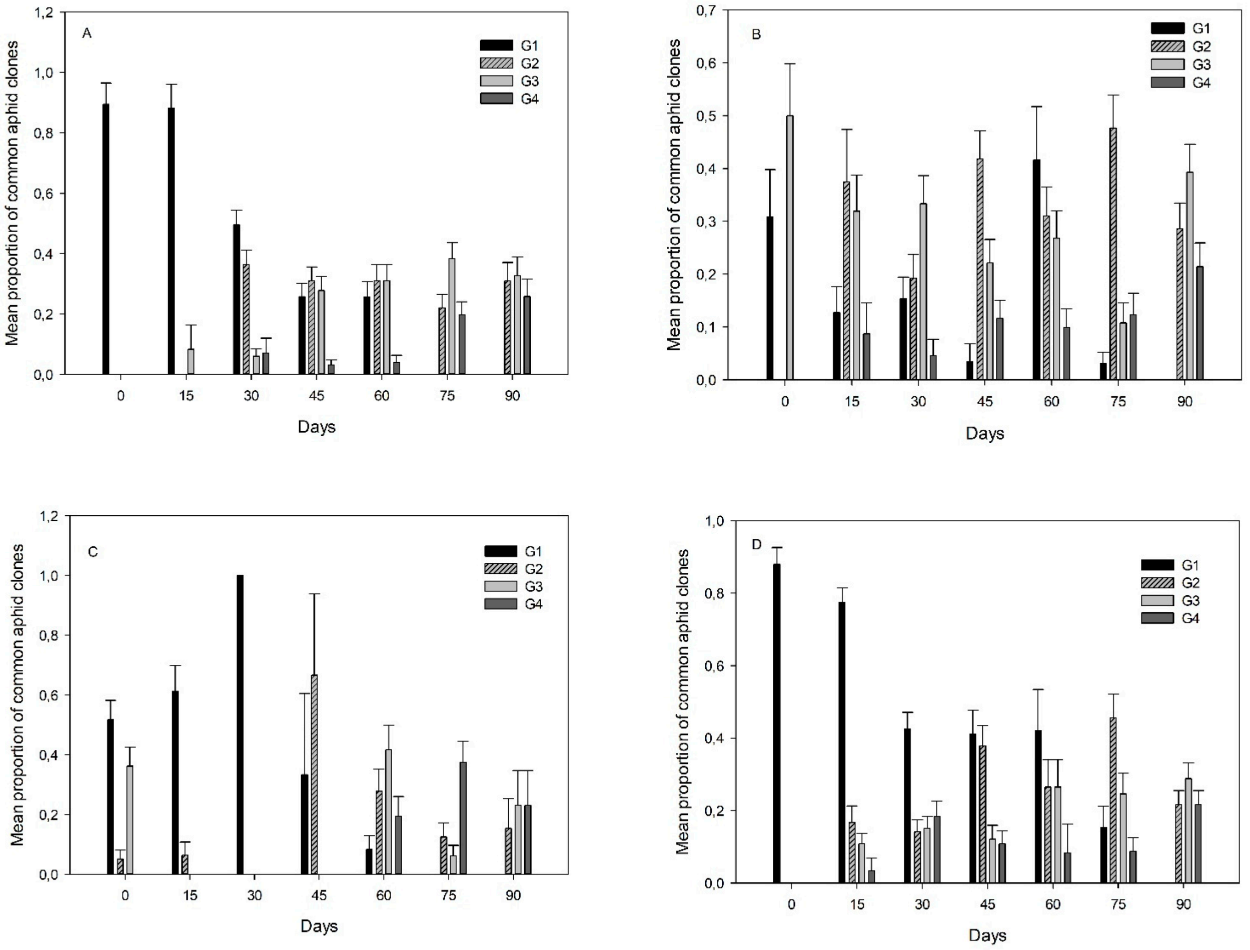

Figure S2. Predominance of common aphid clones in the different localities studied: A) Llastuco, B) Mafil, C) Pichoy and D) Santa-elvira across the season.

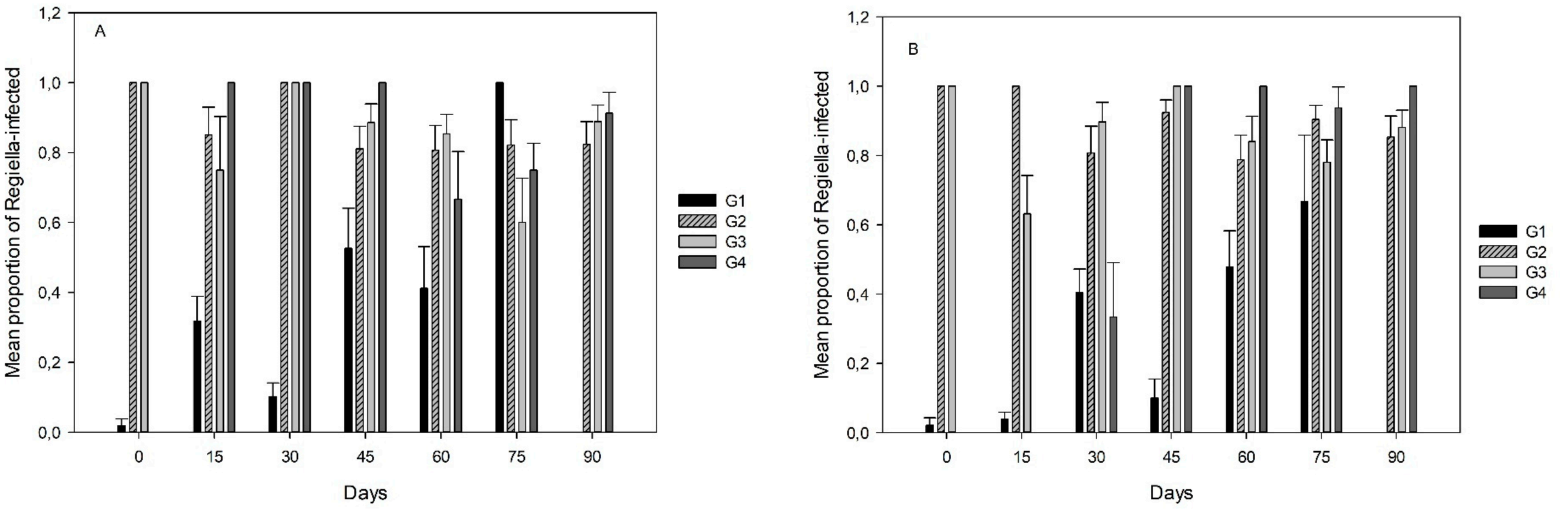

Figure S3. Mean proportion (±SE) of infected aphids with the common endosymbiont *R. insecticola* in the different aphid clones studied (G1, G2, G3 and G4) on A) wheat and B) oat across one season.
